# Neurogenic dysphagia in traditional Chinese medicine

**DOI:** 10.1002/brb3.1812

**Published:** 2020-09-18

**Authors:** Jia Liu, Lu‐ning Wang

**Affiliations:** ^1^ Department of Neurology Xuanwu Hospital Capital Medical University Beijing China; ^2^ Department of Geriatric Neurology Chinese PLA General Hospital Beijing China

**Keywords:** Chinese traditional medicine, neurogenic dysphagia, pathophysiology, therapy

## Abstract

**Background:**

Neurogenic dysphagia is the difficulty in swallowing caused by neurological diseases, which is a very common symptom in neurological disorders. In this paper, we try to summarize the opinions in the pathophysiology and therapy of dysphagia in ancient China (before AD 1840) through the records in the literatures from all the dynasties.

**Methods:**

We searched the databases including Chinese Medical Classics, China National Knowledge Infrastructure, Wanfang Data, MEDLINE, and ISI Proceedings until July 2020, with the search terms “dysphagia” and “difficulty in swallowing” in English and their Chinese equivalents.

**Results and Conclusions:**

The concept of neurogenic dysphagia was first described as ***Hou Bi*** in the Yellow Emperor's Internal Classic, which is the first Chinese medical classic and the origin of traditional Chinese medicine (TCM) theory. In the different eras, the pathogenesis of neurogenic dysphagia was explained mainly by three theories in TCM, that is, the wind‐phlegm blocking collaterals, the deficiency of liver and kidney‐essence, and the Qi‐stagnation with static blood. In addition to the TCM prescriptions, acupuncture is characteristic treatment and seems to be effective. However, the evidences of efficacy and safety from clinical trials are still required.

## Graphical abstract

The concept of neurogenic dysphagia was first described as Hou Bi in the Yellow Emperor's Internal Classic, which is the first Chinese medical classic and origin of traditional Chinese medicine (TCM) theory. In different eras, the pathogenesis of neurogenic dysphagia was explained by three theories in TCM including wind‐phlegm blocking collaterals, deficiency of liver and kidney‐essence, and Qi‐stagnation with static blood. In addition to the TCM prescriptions, acupuncture is characteristic and seems to be effective. However, the evidences of efficacy and safety from clinical trials are required.

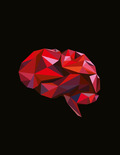

## 1 INTRODUCTION

Neurogenic dysphagia is also called oropharyngeal dysphagia, which is distinct pointing to the difficulty in swallowing caused by the neurological diseases (Buchholz, 1994). Actually, dysphagia is a very common symptom in neurological disorders, such as stroke, neurodegenerative diseases, multiple sclerosis, neuromuscular diseases, and Chiari malformation (Prosiegel, 2007). As far as the neuroanatomy, both of central and peripheral nervous systems are involved in the function of swallow, including cerebral cortex, basal ganglia, brainstem, cerebellum, lower cranial nerves, myenteric ganglia, and neuromuscular junction (Bakheit, 2001). Dysphagia is a fatal symptom, which affects patient's health, quality of life, and nutritional status. In recognition of dysphagia in western medical history, the word “dysphagy” derives from ancient Greek and was first suggested by Nicolatopoulos in 1907 (Megna et al., 2012). The earliest record related to dysphagia could be traced back to the anatomic and physiologic studies of larynx by Galenos (AD 129–199) (Feldmann, 2001). In comparison with western medicine history, records on dysphagia have been detected in thousand‐year's history of traditional Chinese medicine, which could be traced back to Yellow Emperor's Internal Classic, the first Chinese medical classic completed during Warring States Period to Han Dynasty (from 475 BC to AD 220). However, the records on dysphagia are not well known to the international scholars due to the difficulty in understanding ancient Chinese.

There are some basic traditional Chinese medicine (TCM) principles (Liu & Wang, 2017; Liu, Tian, & JZ., 2012; Unschuld, 2003; Yellow, 1963) such as six evils (wind, cold, summer heat, humidity, dryness, and fire) of the nature, which are believed as the pathogens and attack five depots (liver, heart, spleen, lung, and kidney), and six palaces (gallbladder, stomach, small intestine, large intestine, urinary bladder, and triple energizer) to finally cause the diseases. It is well known as the theory of visceral manifestation. The concept of Yin‐Yang [dualism] is an important theory in TCM. The details of the Yin‐Yang categorization of the universe, and of the immediate environment of human existence, include an understanding of human health and illness. The phlegm was regarded as a harmful liquid substance in the body, which can be due to Yin deficiency. Qi is an important TCM concept, which is a flowing energy in the body to ensure the free flow of emotions, blood, and water. The Qi controlling heaven comes down, and the Qi at the fountain joins from below. Collaterals are also known as the meridian in Western acupuncture literature, in which Qi and blood are transported and can be blocked by the phlegm. Pulse diagnosis is an assessment in TCM with the many possible forms of the movement in the vessels. Slippery pulse means dampness or phlegm, retention of food, or even pregnancy. Wiry pulse indicates pain or tension, while hesitant pulse suggests low energy or cold.

## 2 METHODS

In this paper, we aimed to summarize the opinions in the pathophysiology and therapy of dysphagia in ancient China (before AD 1,840) through the records in the literatures from all the dynasties. The databases we searched include Chinese Medical Classics, China National Knowledge Infrastructure, Wanfang Data, MEDLINE, and ISI Proceedings until July 2020, with the search terms “dysphagia” and “difficulty in swallowing” in English and their Chinese equivalents.

## 3 RESULTS AND DISCUSSION

In TCM, neurogenic dysphagia was usually mentioned as “喉痹[***Hou Bi*]**”, while esophageal dysphagia was called “噎嗝[***Ye Ge***]”. Therefore, the differences in the two subtypes of dysphagia have been already recognized in ancient times. The earliest record of neurogenic dysphagia was located in Yellow Emperor's Internal Classic, which is acknowledged as the first Chinese medical classic (from 475 BC to AD 220; Liu & Wang, 2017; Liu et al., 2012; Unschuld, 2003; Yellow, 1963), as well as the origin of TCM theory. There are six evils attack five depots and six palaces to finally cause the diseases. Qi is an important TCM concept, which is a flowing energy in the body to ensure the free flow of emotions, blood, and water. As far as the pathophysiology of dysphagia in TCM, three opinions can be summarized, that is, the wind‐phlegm blocking collaterals, the deficiency of liver and kidney‐essence, and the Qi‐stagnation with static blood.

In Han Dynasty or even earlier, the exogenous wind stirring was believed as the main cause for neurogenic dysphagia with slippery pulse. For instance, neurogenic dysphagia was attributed to the exogenous wind stirring in together with the deficiency of Qi in Yellow Emperor's Internal Classic (Yellow, 1963). Interestingly, the Chinese word of “stroke” was first mentioned in Han Dynasty by Zhang Zhongjing (AD 150–219), which meant being attacked by the exogenous wind (Zhang, 2008). As a frequent secondary symptom due to stroke, neurogenic dysphagia was thought to result from the same mechanism. Sun Simiao in Tang Dynasty (AD 581–682) described a male patient with limb paralysis, dysphagia, and aphasia, who was most likely to suffer stroke (Sun, 1995). He speculated the symptoms were caused by the wind attacking depots and palaces. This theory was developed by Zhu Danxi (AD 1281–1358) in Yuan Dynasty (Zhu, 2008). The concrete mechanisms were the exogenous wind stirring due to suffering the deficiency of Qi, spleen deficiency of Qi producing phlegm, and finally the wind‐phlegm blocking collaterals. Therefore, the therapy was targeted to the wind‐phlegm in TCM.

The deficiency of liver and kidney‐essence is another important theory to explain neurogenic dysphagia with wiry pulse. It was also first mentioned in Yellow Emperor's Internal Classic (Yellow, 1963). There were fundamental theories noted like “spirit brilliance originates from the heart” and “kidney generates water liquid.” In Song Dynasty, Liu Wansu (AD 1118–1200) thought the deficiency of kidney‐essence would generate the insufficient water liquid to against heart fire, thereafter affect the brain function and cause the symptoms like dysphagia (Liu, 2005). Furthermore, the endogenous wind could be produced by the liver under the case of deficiency of liver and kidney‐essence, and attack depots and palaces (Li, 2011). Neurogenic dysphagia is not rare in neurodegeneration like Parkinson's disease, while Parkinson's disease was also attributed to the endogenous wind stirring in TCM as “wind shaking” (Zhang, Dong, & Román, 2006). Therefore, nourishing the liver and kidney to eliminate the wind is a potential therapeutic target for neurogenic dysphagia in TCM.

The Qi‐stagnation with static blood is a distinct theory for neurogenic dysphagia with hesitant pulse and is different from the exogenous and endogenous wind theory. In Ming Dynasty, Yin deficiency was emphasized as the mechanism of neurogenic dysphagia by Zhang Jingyue (AD 1768–1831; Zhang, 1991). The imbalance of Yin‐Yang was due to Yin deficiency, which would lead to the Qi‐stagnation with static blood and the symptoms like dysphagia. He believed the phlegm was produced due to Yin deficiency. The treatment should focus on nourishing Yin, rather than eliminating the phlegm. In Qing Dynasty, Shen Jin'ao (AD 1768–1831) wrote in the book “Za Bing Yuan Liu Xi Zhu” that obese people were prone to suffering neurogenic dysphagia and stroke, because of the Qi‐stagnation and static blood (Shen, 1994). Therefore, the Qi and blood circulation should be improved and regarded as the therapeutic target. In addition to TCM prescriptions, acupuncture was thought to be an effective treatment (Chan et al., 2012; Geeganage, Beavan, & Ellender, 2012), such as body, tongue, scalp acupuncture, and bloodletting puncture.

## 4 CONCLUSIONS

In summary, the concept of neurogenic dysphagia was first described as ***Hou Bi*** in the Yellow Emperor's Internal Classic, which is the first Chinese medical classic and the origin of TCM theory. In the different eras, the pathogenesis of neurogenic dysphagia was explained mainly by three theories in TCM, that is, the wind‐phlegm blocking collaterals, the deficiency of liver and kidney‐essence, and the Qi‐stagnation with static blood. In addition to the TCM prescriptions, acupuncture is characteristic treatment and seems to be effective. However, the evidences of efficacy and safety from clinical trials are still required.

## CONFLICT OF INTEREST

The authors confirm that this article content has no conflict of interest.

## AUTHOR CONTRIBUTION

Drs. Liu and Wang involved in study concept and design, Drs. Liu and Wang performed searching for the literatures, Dr. Liu wrote the manuscript, Drs. Liu and Wang involved in critical revision of the manuscript for important intellectual content.

## ETHICAL APPROVAL

Not available.

### Peer Review

The peer review history for this article is available at https://publons.com/publon/10.1002/brb3.1812.

## Data Availability

Data sharing is not applicable to this article as no new data were created or analyzed in this study.
